# An empirical study on the relationship between emotional labor and work performance among university teachers

**DOI:** 10.3389/fpsyg.2024.1470436

**Published:** 2024-10-07

**Authors:** Danna Hao

**Affiliations:** College of Education Science, Weinan Normal University, Weinan, China

**Keywords:** university teachers, emotional labor, work performance, empirical study, emotional event theory

## Abstract

This study is based on the theory of emotional events and explores the relationship between emotional labor and the job performance of university teachers. Relevant data was obtained through a questionnaire, and a structural equation model was constructed for path analysis. The study found that the surface performance of university teachers has a significant negative impact on relationship performance and task performance; deep performance and proactive authenticity of university teachers have a significant positive impact on relationship performance, task performance, and adaptability performance, respectively; the passive reality of university teachers has a significant negative impact on relationship performance, task performance, and adaptability performance. Based on research findings, this study proposes suggestions for the performance evaluation mechanism of university teachers, including improving the recruitment methods, increasing the emotional labor assessment and incentive mechanism, focusing on alleviating negative emotions of teachers, strengthening the awareness guidance and education of school management and teachers on emotional labor, establishing training courses on emotional labor for teachers, arranging various activities reasonably, and creating a good working atmosphere. Ultimately, these suggestions aim to help university teachers recognize the significance of emotional labor, promote their educational and teaching work, and provide new methods and paths for improving the work performance of university teachers.

## 1 Introduction

As an important carrier of knowledge innovation and dissemination, university teachers are the most important cornerstone of the higher education system. In the normal teaching and research process of university teachers, they often need to express emotions as required to achieve teaching goals, that is, to engage in emotional labor. College teachers, as natural persons, may experience personal emotions at any time during the teaching process. Unstable emotional labor can have an impact on their physical and mental health, and even seriously affect their work performance. Therefore, as the main body of highly emotional workers, how to maintain the stability of teachers’ labor emotions to promote the continuous development of teachers’ work performance has attracted much attention. From an academic research perspective, previous studies on job performance have mostly focused on task performance and relational performance, while relatively little attention has been paid to the role of emotional labor in job performance. However, an increasing number of studies indicate that emotional labor has a significant impact on job performance in areas such as the service industry. The work of university teachers has a service-oriented nature to a certain extent, and their emotional labor may play an important role in performance indicators such as teaching effectiveness, student evaluation, and research output.

From an academic research perspective, early studies on emotional labor originated from a focus on service industry personnel (Jing, 2009), where organizations generally required employees to be friendly and smiling toward service recipients. As an important place for knowledge innovation and talent cultivation, universities have constantly raised their requirements for teachers. College teachers not only need to impart professional knowledge, but also pay attention to the comprehensive development of students, and engage in effective communication and interaction with students, colleagues, leaders, and others. With the updating of educational concepts, the emphasis on student-centered education requires teachers to be able to keenly perceive and respond to students’ emotional needs, and create a positive learning atmosphere. This requires teachers to invest a significant amount of emotional resources and engage in emotional labor in their work. At the same time, universities are facing a fiercely competitive environment, including enrollment competition, research achievement competition, and so on. Teachers are under significant work pressure and need to maintain good work status and emotional performance in various tasks and challenges. Research has found that emotional labor can affect people’s mental health (Hongxia, 2010), affect the relationship between teachers and students (Jianfeng and Xinbo, 2017), and affect innovative work performance (Shengnan, 2019). Hwang and Park’s (2022) study found that different manifestations of emotional labor have varying degrees of impact on work performance. Therefore, in school teaching, the interactivity of teachers’ teaching processes, emotional expression, and emotional influence have a significant impact on students.

Based on the above discussion, previous research on job performance has mainly focused on task performance and relationship performance, while relatively less attention has been paid to the role of emotional labor in job performance. However, an increasing number of studies indicate that emotional labor has a significant impact on job performance in fields such as the service industry. The work of university teachers to some extent has a service-oriented nature, and their emotional labor may play an important role in performance indicators such as teaching effectiveness, student evaluation, and research output. In addition, in the current era of digitization and globalization, university teachers are facing a more diverse student population and a more complex educational environment, and the ways and strategies of emotional labor may also change. Therefore, an in-depth exploration of the relationship between emotional labor and the job performance of university teachers is of great practical significance and urgency for understanding the work characteristics of university teachers, optimizing teacher management, and improving educational quality. This article aims to explore in depth the impact of emotional labor on the work of university teachers, to provide a reference for schools to propose better strategies for the performance management of university teachers.

## 2 Literature review

### 2.1 Theoretical basis

The theory of emotional events was proposed by Weiss and Cropanzano. This theory uses the chain of “event emotion attitude-behavior” to explain the mechanism by which individual emotions affect work processes and outcomes (Weiss, 1996). This theory proposes that emotional events in the workplace can be divided into two types: negative and positive (Brotheridge, 2004). Based on the theory of emotional events, scholars have conducted empirical research on the relationship between emotional commitment, organizational support, emotional labor, and job satisfaction among teachers (Andrew Richards et al., 2020). This study applies the theory of emotional events to further illustrate how the emotions of university teachers, under the influence of emotional events (emotional labor), will affect their work performance.

### 2.2 Definition and dimensional definition of emotional labor

#### 2.2.1 Definition of emotional labor

Emotional labor is the process of modifying emotions to gain rewards, express the required emotions or feelings of work through bodily expressions (Hochschild, 1983). Ashforth and Humphrey (1993) consider emotional labor as an outward behavioral manifestation, which, influenced by external factors such as the environment and individuals, can have different effects on employee emotions. Emotional labor involves continuously adjusting and restraining emotions to display the expected emotional state of the organization during work and communication, with sources of emotional influences on employees stemming from their environment. Emotional labor refers to the behavior exhibited by employees when interacting with internal colleagues or external consumers, where they restrain or mediate their authentic emotions for related benefits (Jones, 1999). The focus of emotional labor lies in the psychological processes of individuals, involving self-regulation and adjustment of emotions in certain situations within the organization, aiming to align one’s emotions with the organization’s requirements, which is an ongoing process of adjustment and correction (Diefendorff and Gosserand, 2003). Chunxing (2005) suggests that emotional labor is the continuous adjustment of individual emotions within an organization to meet organizational needs, a process of emotional regulation that can influence the emotions of others and contribute to achieving organizational goals, views emotional labor as the integration of individual emotional expression with organizational needs, considering it a necessary process for communication. Chaoying and Lili (2011) argue that employees within organizations continuously adjust their emotions to meet the overall emotional needs of the organization due to the nature of their work. Grandey and Melloy’s (2017) emotional labor model summarizes previous experiences, further describes and analyzes the complete process and mechanism of emotional labor, and further clarifies that emotional labor is the process of emotional regulation carried out to achieve interpersonal interaction goals. Refers to an individual’s complex physiological evaluation and experience of something, including their connections with other individuals. It emphasizes inner and stable psychological experiences (Xiaoan and Han, 2021). Jianwei et al. (2021) proposed that emotional labor is a dynamic process in which the labor subject consciously regulates and manages their own emotions to achieve the goal of “exchange” to effectively achieve their goals.

In summary, this paper defines emotional labor as the expression of emotions by individuals during labor processes to meet organizational requirements, facilitating individual development toward organizational goals, and exhibiting emotional behavior to meet organizational expectations and customer demands.

#### 2.2.2 Dimensional definition of emotional labor

There are various interpretations of the content of emotional labor in existing studies, resulting in different dimensional perspectives. However, scholars generally agree that emotional labor is a multidimensional and multi-component concept. Hochschild (1983) identifies three dimensions of emotional labor: surface performance, proactive deep acting, and passive deep acting. Ashforth and Humphrey (1993) categorize emotional labor into surface performance, deep acting, and authentic emotions. Wharton and Erickson (1993) classify emotional labor into positive, negative, and neutral emotions. Morris and Feldman (1996) propose a four-dimensional theory of emotional labor, which includes the frequency of emotional labor, the level or degree of emotional expression, emotional demands at work, and the coordination between authentic emotions and organizational needs. Zapf and Holz (2006) suggests that emotional labor can be divided into the frequency of positive emotional expression, diversity of emotions in different scenarios, sensitivity to customer emotions, perspective-taking, emotional control, emotional expression driven by organizational needs, and interactive emotional control. Kruml and Geddes (2000) distinguish emotional labor into emotional effort and emotional dissonance. Diefendorff and Gosserand (2003) categorize emotional labor into surface performance, deep acting, and expression of authentic emotions, with five items under surface performance, six items under deep acting, and three items under expression of authentic emotions. Grandey (2003) categorizes emotional labor into surface performance and deep acting. Brotheridge (2002)divides emotional labor into the dimensions of emotional dissonance with 14 items and emotional effort with 5 items. Yanling (2007) categorizes emotional labor into surface behavior, active deep behavior, and passive deep behavior.

Based on the above discussion, this paper divides emotional labor into four dimensions: surface performance, deep acting, proactive authenticity expression, and passive authenticity expression. Surface performance, proactive authenticity expression, and passive authenticity expression draw from the scale developed by Diefendorff and Gosserand (2003), while the deep acting dimension adopts the scale developed by Taiwanese scholar Wu Peijun based on Grandey (2003), which includes six items. The descriptions of the items have been adjusted appropriately based on the research content. Surface performance refers to the expression of emotions in line with organizational requirements by changing external expressions or bodily language when an individual’s emotions conflict with those expected by the organization, representing a pretense; deep acting involves individuals actively controlling their emotions through emotional regulation to meet organizational emotional needs when their emotions conflict with those of the organization; proactive authenticity expression refers to the natural expression of emotions by individuals when their emotions align with organizational expectations. Passive authenticity expression occurs when individuals exhibit positive emotions required by the organization due to the influence of the surrounding environment, representing a direct response to positive environmental stimuli.

### 2.3 Definition and dimensional definition of work performance

#### 2.3.1 Definition of work performance

Campbell et al. (1990) views work performance as the behaviors exhibited by employees to achieve organizational goals. Borman and Motowidle (1993) argue that work performance refers to the behaviors exhibited by organizational members in the process of work that are aligned with organizational goals and contribute to achieving these goals. These behaviors can be measured externally and evaluated based on the amount of contribution. Brumbrach (1988) suggests that performance consists of behaviors and results, where behaviors represent the degree of completion. Zeyan (2002) defines work performance as the process of evaluating and considering the behaviors, performances, and results of organizational members, the work performance as the content completed by organizational members within a specified time frame. Mathew et al. (2012) also argue through research that performance is a comprehensive assessment of individual behaviors and results.

In summary, this study adopts a comprehensive interpretation of performance and defines work performance as the actual behaviors exhibited by teachers to practice and achieve teaching goals, research tasks, social services, and other objectives, aligning individual actions with organizational goals and translating them into actions in practice.

#### 2.3.2 Dimensional definition of work performance

Johnson (2001) further specifies adaptability performance as the proficiency level at which employees adjust their behavior in the face of changes in the environment, events, or new occupations. Jianmin and Changquan (2006) propose three dimensions of performance, including task performance, personal trait performance, and interpersonal relationship performance. Zhitong (2013) confirms through exploratory factor analysis that university teachers’ work performance includes three dimensions: quality performance, innovation performance, and learning performance. Jianfeng and Xinbo (2017) suggest that research-oriented university teachers’ work performance mainly includes task performance and contextual performance. Cao Zhifeng divides work performance into task performance, relationship performance, and adaptability performance. This study adopts Cao Zhifeng’s viewpoint and divides work performance into three dimensions with a total of 20 items. Items 1–10 represent task performance, items 11–15 represent relationship performance, and items 16–20 represent adaptability performance.

### 2.4 Emotional labor and work performance

Adelmann (1995) found through empirical studies that emotional labor does not hurt, harm individuals’ physical and mental health. Morris and Feldman (1997) found that emotional instability and incongruence can lead to feelings of fatigue, thereby affecting work performance; increased emotional labor over time requires individuals to internalize more. Abraham (1999) demonstrated through research that emotional dissonance is negatively correlated with organizational commitment and work performance. Zapf and Holz (2006) found that positive emotional expression is positively correlated with performance and personal achievement. Totterdell and Holman (2003) found that expressing emotionally exhausting emotions can lead to emotional exhaustion in individuals, while the issuance of positive emotions has a positive promoting effect on organizational performance. Liu et al. (2004) found through research that emotional labor can lead to tense behaviors and feelings in the workplace. Brotheridge and Grandy (2002) found that work efficiency and satisfaction are influenced by emotional expression; positive emotions promote an individual’s sense of achievement in work. Ashforth and Humphrey (1993) proposed through research that being adept at controlling one’s emotions at work can enhance employees’ work performance and ensure higher profits. Forced adaptation of emotional labor can reduce work performance (Fangmin, 2009). Emotional labor can cause work fatigue in employees, thereby affecting their work performance (Lihong, 2012). Qi and Yaoyu (2012) studied from the perspective of job satisfaction and found that emotional labor hurts, harms employees’ job satisfaction, which in turn affects customer satisfaction, and thus affects the achievement of organizational goals and organizational performance. Kim et al. (2017) analyzed the relationship between emotional labor and work performance using airline employees as an example, and explored the moderating role of perceived leadership, colleague, and organizational support in the relationship between emotional labor and work performance. It was found that the relationship between the two was significant, and perceived leadership and colleague support enhanced the positive correlation between deep acting and work performance, while perceived leadership support also exacerbated the negative correlation between surface performance and work performance. Heydari et al. (2020) found that more emotional labor can lead to more emotional fatigue among employees and reduce their job satisfaction. Heydari et al. (2020) found that to improve employee satisfaction and performance, appropriate employee training and other measures can be taken to increase employee income, thereby enhancing their performance. Hwang and Park (2022) explored the relationship between emotional labor, job satisfaction, and work performance among nurses and found that surface performance is weakly correlated with work performance, while deep acting significantly predicts work performance positively. Yingji (2022) found that individuals with high emotional labor can autonomously regulate and control their emotions, and compared to individuals with passive emotional labor, those with high emotional labor have higher psychological resilience after self-motivation and therefore perform better at work. Aixiang (2023) found that employees’ emotional labor affects their work performance based on continuously adjusting their states and emotions to guide them into work more rationally. Suzuki and Pitts (2023) believe that positive emotional expression and effective psychological support and intervention can help students discover more potential and achieve more success. Wibowo et al. (2024) found that sincere psychological support and emotional expression can help athletes develop psychological resilience and achieve better results in competitions. Stylos et al. (2024) demonstrated the crucial role of emotional responses and emotional labor in different life experiences or events.

**Figure 1:**
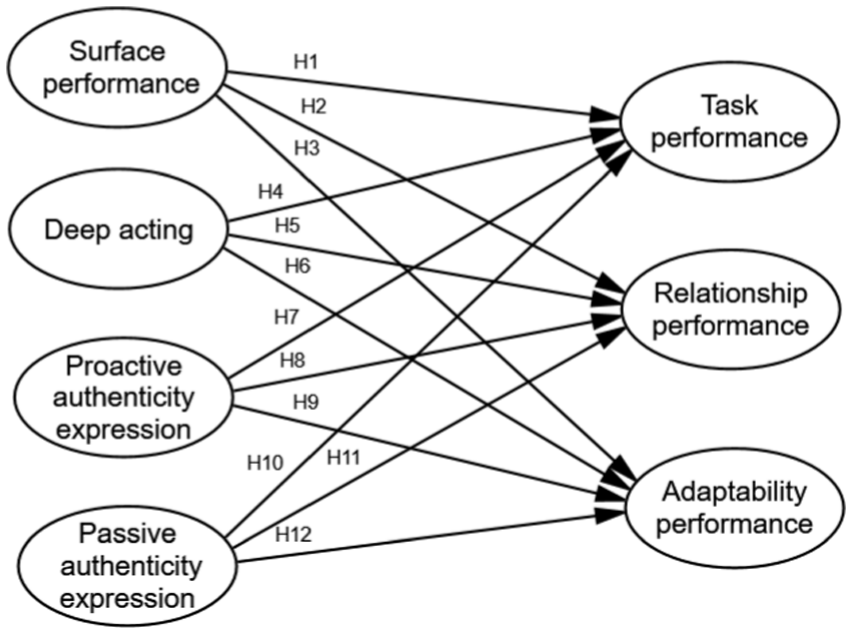
A Research model on the impact of emotional labor on job performance.

Based on the above discussions, this study proposes the following hypotheses (Figure 1):

*H1*: The surface performance of university teachers hurts, harms task performance in work performance.

*H2*: Surface performance of university teachers has a negative impact on relationship performance in work performance.

*H3*: Surface performance of university teachers has a negative impact on adaptability performance in work performance.

*H4*: Deep acting of young teachers has a positive impact on task performance in work performance.

*H5*: Deep acting of university teachers has a positive impact on relationship performance in work performance.

*H6*: Deep acting of university teachers has a positive impact on adaptability performance in work performance.

*H7*: Proactive authenticity expression of university teachers has a positive impact on task performance in work performance.

*H8*: Proactive authenticity expression of university teachers has a positive impact on relationship performance in work performance.

*H9*: Proactive authenticity expression of university teachers has a positive impact on adaptability performance in work performance.

*H10*: Passive authenticity expression of university teachers has a negative impact on task performance in work performance.

*H11*: Passive authenticity expression of university teachers has a negative impact on relationship performance in work performance.

*H12*: Passive authenticity expression of university teachers has a negative impact on adaptability performance in work performance.

## 3 Research methodology

### 3.1 Research subjects

This study primarily focuses on the impact of emotional labor on the performance of university teachers. By analyzing the relationship between different aspects of teachers’ emotional labor and performance, it aims to provide reference opinions for universities in mobilizing teachers’ emotional labor. This study used convenience sampling to distribute questionnaires. Convenience sampling is a non-probability, non-probability sampling survey method that allows researchers to randomly select samples from colleagues and friends they can come into contact with based on their convenience (Coleman, 2009). Through this method, it is relatively easy and cost-effective to find in-service teachers in universities, thus achieving the survey objectives of this study. The main sample sources include teachers from affiliated universities, provincial universities, and private universities. Minglong (2009) believes that in general, a sample size greater than 200 can be considered a medium-sized sample. To pursue stable SEM analysis results, it is best to have a sample size of 200 or more. This study distributed a total of 300 questionnaires, with 255 returned and 241 deemed valid, resulting in an effective questionnaire rate of 94.50%. Among the respondents, there were 84 males and 157 females, 113 undergraduates, 92 master’s students, and 36 doctoral students or above. Additionally, there were 91 individuals under 25 years old, 54 aged between 26 and 35, 63 aged between 36 and 45, 26 aged between 46 and 55, and 7 aged 56 or above (Table 1). For the sake of convenience in the study, surface performance is represented as BCBX, deep acting as SCBX, proactive authenticity expression as ZDZS, passive authenticity expression as BDZS, task performance as RWJX, relationship performance as GXJX, and adaptability performance as SYX (Table 1).

**Table 1:** Descriptive statistics.

Category	Concrete content	Number of people	Percentage (%)
Gender	Males	84	34.85
	Females	157	65.15
Education	Undergraduates	113	46.89
	Master’s	92	38.17
	Doctor	36	14.94
Age	Under 25 years old	91	37.75
	Between 26 and 35	54	22.41
	Between 36 and 45	63	26.14
	Between 46 and 55	26	10.79
	56 or above	7	2.90

### 3.2 Research method

This study adopts a quantitative analysis approach, uses SPSS 26.0 and AMOS 26.0 statistical analysis software tools to preprocess and statistically analyze the collected questionnaire data, acquiring relevant data through questionnaires. Exploratory factor analysis is employed to test the reliability and validity of all measurement tools, thereby verifying the practicality and effectiveness of statistical analysis software in this study. Subsequently, the analysis of composite reliability and discriminant validity is conducted to examine the correlation of the data. Finally, a structural equation model is established to validate the research hypotheses.

## 4 Research findings

The overall reliability coefficient of the scale is 0.915. Based on the CITC value of 0.4, items with CITC values below this threshold are removed. Specifically, item BCBX2 under the surface performance dimension has a CITC value of 0.388, item SCBX3 under the deep acting dimension has a CITC value of 0.383, and item GXJX3 under the relationship performance dimension has a CITC value of −0.005. After removing items with CITC values below 0.4, the reliability coefficients for each dimension are as follows: surface performance dimension 0.821, proactive authenticity expression dimension 0.767, passive authenticity expression dimension 0.834, deep acting dimension 0.767, task performance dimension 0.869, relationship performance dimension 0.717, and adaptability performance dimension 0.808. The KMO measure is 0.895, and Bartlett’s test of sphericity yields a significance probability of 0.000, indicating that all scales have acceptable reliability and validity coefficients, thus ensuring the questionnaire’s reliability (Table 2). These results suggest significant differences between items within dimensions, making them suitable for factor analysis (Table 2).

**Table 2:** Reliability and validity test.

Factor	Item	Corrected item-total correlation	Cronbach’s alpha if item deleted	Cronbach’s alpha	KMO
BCBX (surface performance)	BCBX1	0.477	0.812	0.821	0.895
	BCBX3	0.561	0.798		
	BCBX4	0.677	0.778		
	BCBX5	0.641	0.784		
	BCBX6	0.621	0.788		
	BCBX7	0.581	0.795		
ZDZS (proactive authenticity expression)	ZDZS1	0.513	0.818	0.767	
	ZDZS2	0.635	0.657		
	ZDZS3	0.688	0.598		
BDZS (passive authenticity expression)	BDZS1	0.727	0.738	0.834	
	BDZS2	0.678	0.785		
	BDZS3	0.682	0.783		
SCBX (deep acting)	SCBX1	0.578	0.715	0.767	
	SCBX2	0.445	0.750		
	SCBX4	0.566	0.720		
	SCBX5	0.640	0.702		
	SCBX6	0.496	0.737		
RWJX (task performance)	RWJX1	0.656	0.851	0.869	
	RWJX2	0.561	0.859		
	RWJX3	0.537	0.861		
	RWJX4	0.613	0.857		
	RWJX5	0.628	0.855		
	RWJX6	0.567	0.858		
	RWJX7	0.658	0.852		
	RWJX8	0.487	0.869		
	RWJX9	0.658	0.851		
	RWJX10	0.613	0.855		
GXJX (relationship performance)	GXJX1	0.411	0.418	0.717	
	GXJX2	0.421	0.424		
	GXJX4	0.453	0.415		
	GXJX5	0.420	0.436		
SYX (adaptability performance)	SYX1	0.494	0.805	0.808	
	SYX2	0.678	0.745		
	SYX3	0.649	0.755		
	SYX4	0.677	0.746		
	SYX5	0.497	0.800		

Exploratory factor analysis is conducted on each variable, and based on the total variance decomposition table, the eigenvalues of the first 7 principal components are all greater than 1, while those from the 8th onward are less than 1. Furthermore, the cumulative contribution rate of the first 7 principal components is 66.306%.

Finally, as the factor loadings of the 6th item in the task performance dimension and the 2nd item in the relationship performance dimension are both less than 0.5, these two items are deleted, resulting in the remaining 34 items (Table 3).

**Table 3:** Results of factor analysis.

	BCBX	ZDZS	BDZS	SCBX	RWJX	GXJX	SYX
BCBX1	0.560						
BCBX3	0.845						
BCBX4	0.716						
BCBX5	0.595						
BCBX6	0.839						
BCBX7	0.837						
ZDZS1		0.711					
ZDZS2		0.657					
ZDZS3		0.790					
BDZS1			0.650				
BDZS2			0.739				
BDZS3			0.581				
SCBX1				0.560			
SCBX2				0.504			
SCBX4				0.695			
SCBX5				0.793			
SCBX6				0.628			
RWJX1					0.583		
RWJX2					0.693		
RWJX3					0.562		
RWJX4					0.728		
RWJX5					0.734		
RWJX7					0.732		
RWJX8					0.764		
RWJX9					0.742		
RWJX10					0.679		
GXJX1						0.632	
GXJX4						0.651	
GXJX5						0.777	
SYX1							0.591
SYX2							0.632
SYX3							0.539
SYX4							0.553
SYX5							0.653

Surface performance is represented as BCBX, deep acting as SCBX, proactive authenticity expression as ZDZS, passive authenticity expression as BDZS, task performance as RWJX, relationship performance as GXJX, and adaptability performance as SYX.

Based on the output data, the composite reliability (CR) values were calculated to assess the internal consistency of the indicators of the intrinsic motivation and task performance dimensions, as well as the discriminant validity between them. The composite reliability (CR) value represents the combination of the reliability of all measured variables, indicating the internal consistency of the constructs. A higher CR value suggests greater internal consistency of the construct. Fornell and Larcker suggested that the acceptable threshold for average variance extracted (AVE) should fall between 0.36 and 0.5. In this study, all AVE values met the standard, and CR values were all greater than 0.7, indicating good test results (Table 4).

**Table 4:** Composition reliability and discriminatory validity.

Factor	Average variance extracted (AVE) value	Composite reliability (CR) value
BCBX (surface performance)	0.472	0.838
ZDZS (proactive authenticity expression)	0.599	0.813
BDZS (passive authenticity expression)	0.648	0.846
SCBX (deep acting)	0.493	0.827
RWJX (task performance)	0.460	0.879
GXJX (relationship performance)	0.402	0.759
SYX (adaptability performance)	0.515	0.840

**Figure 2:**
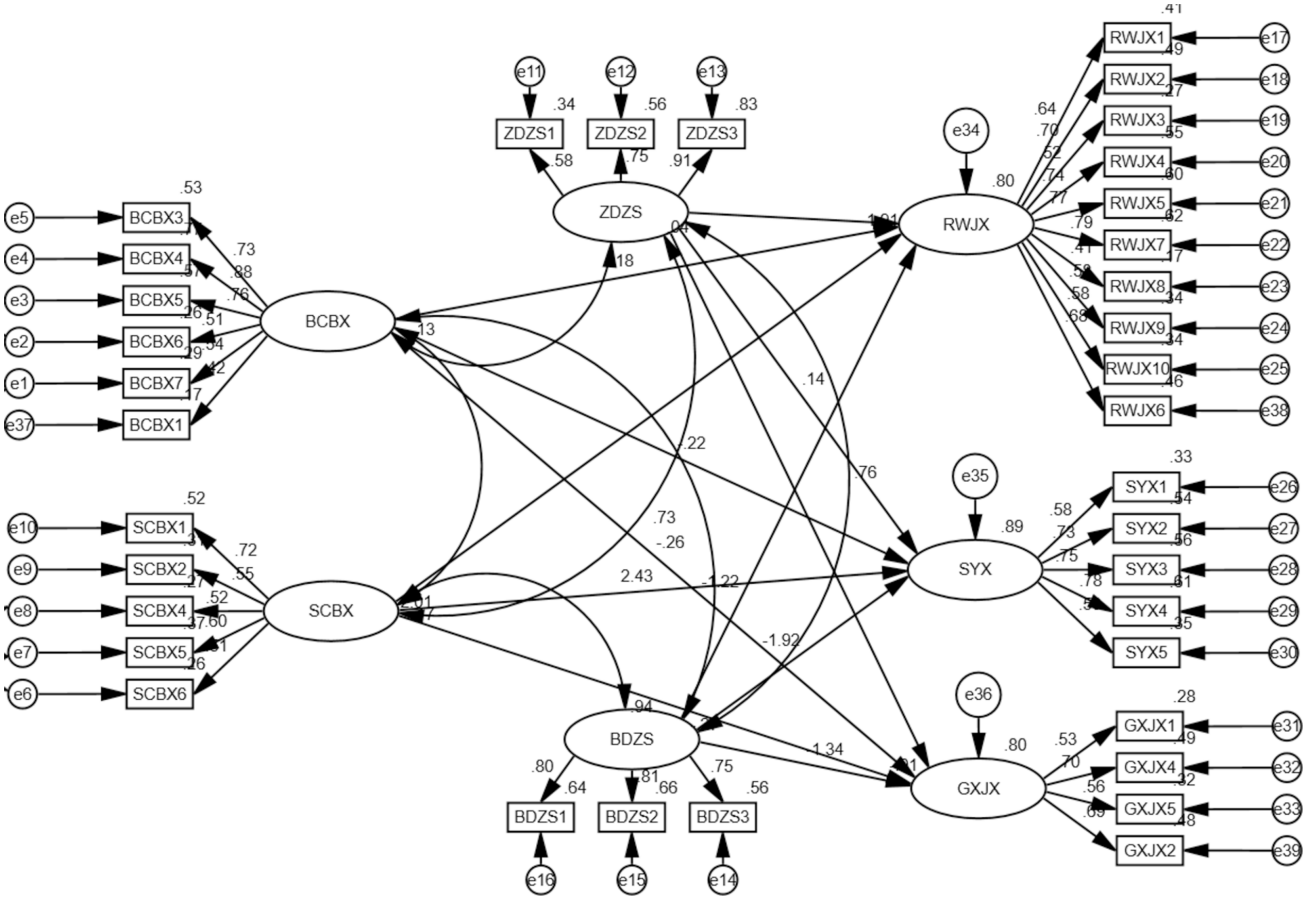
Hypothesis test. Notes: Surface performance is represented as BCBX, deep acting as SCBX, proactive authenticity expression as ZDZS, passive authenticity expression as BDZS, task performance as RWJX, relationship performance as GXJX, and adaptability performance as SYX.

A structural equation model was constructed, with the following fit index values: goodness of fit index (GFI) = 0.911, adjusted goodness of fit index (AGFI) = 0.914, normed fit index (NFI) = 0.933, and root mean square error of approximation (RMSEA) = 0.062, which is below the threshold of 0.08, indicating that the model is acceptable (Figure 2).

Through the study, it was found that surface performance (BCBX) has a *p*-value less than 0.05 on task performance (RWJX), with a path coefficient of −0.171, indicating a significant negative effect. Surface performance (BCBX) on adaptability performance (SYX) has a *p*-value more than 0.05, with a path coefficient of −0.153, Indicating that the hypothesis is not valid. Surface performance (BCBX) on relationship performance (GXJX) has a *p*-value less than 0.05, with a path coefficient of −0.218, indicating a significant negative effect. Deep acting (SCBX) on task performance (RWJX) has a *p*-value less than 0.05, with a path coefficient of 0.718, indicating a significant positive effect. Deep acting (SCBX) on adaptability performance (SYX) has a *p*-value less than 0.05, with a path coefficient of 0.581, indicating a significant positive effect. Deep acting (SCBX) on relationship performance (GXJX) has a *p*-value less than 0.05, with a path coefficient of 0.568, indicating a significant positive effect. Proactive authenticity (ZDZS) on task performance (RWJX) has a *p*-value less than 0.05, with a path coefficient of 0.537, indicating a significant positive effect. Proactive authenticity (ZDZS) on adaptability performance (SYX) has a *p*-value less than 0.05, with a path coefficient of 0.484, indicating a significant positive effect. Proactive authenticity (ZDZS) on relationship performance (GXJX) has a *p*-value less than 0.05, with a path coefficient of 0.415, indicating a significant positive effect. Passive authenticity (BDZS) on task performance (RWJX) has a *p*-value less than 0.05, with a path coefficient of −0.651, indicating a significant negative effect. Passive authenticity (BDZS) on adaptability performance (SYX) has a *p*-value less than 0.05, with a path coefficient of −0.535, indicating a significant negative effect. Passive authenticity (BDZS) on relationship performance (GXJX) has a *p*-value less than 0.05, with a path coefficient of −0.546, indicating a significant negative effect (Table 5).

**Table 5:** Research hypothesis test results.

			Estimate	S.E.	C.R.	P	
RWJX	<−--	ZDZS	0.537	0.094	5.717	0.000	True
SYX	<−--	ZDZS	0.484	0.084	4.934	0.000	True
GXJX	<−--	ZDZS	0.415	0.074	3.270	0.001	True
RWJX	<−--	BCBX	−0.171	0.102	−2.191	0.028	True
SYX	<−--	BCBX	−0.153	0.085	−1.918	0.055	False
GXJX	<−--	BCBX	−0.218	0.071	−2.217	0.027	True
RWJX	<−--	SCBX	0.718	0.134	6.597	0.000	True
SYX	<−--	SCBX	0.581	0.113	5.319	0.000	True
GXJX	<−--	SCBX	0.568	0.114	3.704	0.000	True
RWJX	<−--	BDZS	−0.651	0.065	−7.670	0.000	True
SYX	<−--	BDZS	−0.535	0.056	−5.749	0.000	True
GXJX	<−--	BDZS	−0.546	0.063	−4.381	0.000	True

Surface performance is represented as BCBX, deep acting as SCBX, proactive authenticity expression as ZDZS, passive authenticity expression as BDZS, task performance as RWJX, relationship performance as GXJX, and adaptability performance as SYX.

## 5 Analysis and discussion

Based on the data analysis conducted in this study, the following conclusions can be drawn:

(1) Surface performance of emotional labor has a significant negative impact on relationship performance and task performance, while its impact on adaptability performance is not significant. This is consistent with Miner and Miner (2006) viewpoint. This suggests that the surface performance of emotional labor significantly reduces both the relationship performance and task performance of teachers. Teachers may feel emotionally drained, leading to a need to allocate energy, which affects their relationships with others and their ability to accomplish tasks effectively. On the other hand, deep acting in emotional labor has a significant positive impact on relationship performance, task performance, and adaptability performance. Verified Kim’s et al. (2017) viewpoint. This indicates that deep acting in emotional labor significantly improves teachers’ relationship performance, task performance, and adaptability performance. By regulating their emotions in response to job demands, teachers can build better relationships, efficiently complete tasks, and enhance adaptability performance.

(2) Proactive authenticity in emotional labor has a significant positive impact on relationship performance, task performance, and adaptability performance. This is consistent with the research results of Hwang and Park (2022). This implies that demonstrating proactive authenticity significantly improves teachers’ relationship performance, task performance, and adaptability performance. When teachers proactively regulate their emotions and engage in work with their best state of mind, they can effectively enhance relationships with colleagues, accomplish tasks efficiently, and better adapt to job requirements. Conversely, passive authenticity in emotional labor has a significant negative impact on relationship performance, task performance, and adaptability performance. This indicates that passive authenticity in emotional labor reduces teachers’ relationship performance, task performance, and adaptability performance. When teachers display neutral emotions as a result of job demands or external stimuli, rather than expressing their true feelings, it can negatively affect their relationships and their jobs, possibly leading to difficulty in accepting the current situation and impacting work performance.

## 6 Conclusion and recommendations

### 6.1 Improvement of university teachers recruitment process

In the recruitment of teachers, attention should be paid to measuring emotional intelligence, which can be done through psychological assessments. Candidates with higher levels of emotional intelligence and psychological capital should be selected. This study found that individuals with higher surface performance are more likely to experience emotional exhaustion, and their relationship performance is also lower. Therefore, organizations should place significant emphasis on the application of various psychological measurement tools to assess the individual skills and knowledge required for emotional labor. Methods such as group discussions, role-playing, and situational simulations can be used to select suitable employees for related tasks. Additionally, psychological tests should be conducted during the selection process to gain a basic understanding of candidates’ psychological and personality traits. This approach helps identify individuals with traits such as enthusiasm, perseverance, teamwork, and interpersonal skills, which are conducive to emotional labor. By assessing candidates’ emotional intelligence and psychological capital levels, organizations can select applicants with higher emotional labor capabilities, thus effectively enhancing their relationship performance.

### 6.2 Implementing incentive mechanisms for assessing teachers’ emotional labor

Performance evaluation serves as a guiding and directional tool. Therefore, incorporating relevant indicators of emotional labor into the assessment of teachers can effectively encourage them to express the emotions required for their work, thus enhancing their emotional labor capabilities. When assessing teachers’ emotional labor performance, a comprehensive evaluation of their overall qualities should be conducted. Additionally, attention should be given to emotional labor indicators for teachers from different academic backgrounds. Quantitative statistics should be applied to evaluate teachers’ emotional labor, and the results should be linked to rewards and promotion criteria. This approach guides teachers to enhance their ability to autonomously regulate and adjust emotional labor, thereby improving their work performance. Furthermore, it is essential to uphold principles of fairness and impartiality and establish effective measurement indicators.

### 6.3 Addressing teachers’ negative emotions

Negative emotions not only lead to a decline in work performance but also contribute to emotional exhaustion, potentially affecting teachers’ psychological well-being. Therefore, universities should be adept at mitigating teachers’ negative emotions and intervening and guiding them appropriately to reduce the adverse effects of negative emotions. Efforts should be made to actively understand the causes of negative emotions, identify underlying issues, and engage in regular communication with teachers. This communication should focus on understanding the relationships between teachers, work arrangements, and the fairness of performance distribution from the perspective of teachers. Subsequently, targeted adjustments can be made based on actual circumstances.

### 6.4 Strengthening awareness and education on emotional labor for university management and faculty

Currently, many university management teams and faculty members lack sufficient awareness of emotional labor, possibly because they are unaware of the positive and negative effects it can have. It is essential to enhance education and guidance on emotional labor for university management and faculty, making them aware of its importance in teaching, research, and community service. Encouraging the integration of positive emotions into daily work can improve the effectiveness of management across departments and positions, ultimately enhancing work efficiency. Additionally, increasing the understanding of emotional labor among management and faculty helps universities focus on teachers’ emotional and psychological well-being. This, in turn, helps create a conducive work environment and fosters harmonious relationships among colleagues. Assisting management in establishing effective emotional management practices for teachers and raising awareness of emotional labor among faculty members helps them maintain appropriate emotions at work, thereby improving work efficiency. When negative emotions arise, teachers should be equipped to self-regulate effectively, mitigate negative emotions, avoid emotional imbalance, and complete work tasks, establish good working relationships, and better adapt to various work requirements.

### 6.5 Establishing training courses on teacher emotional labor

This study’s analysis suggests that universities should assist teachers in enhancing their ability to express deep acting and proactive authenticity emotions, while minimizing surface performance and passive authenticity emotions. Systematic training programs can be implemented, utilizing methods such as role-playing and perspective-taking exercises to help teachers develop positive relationships with colleagues and students. When negative emotions arise, appropriate coping strategies should be employed to enhance teachers’ management and control of emotional labor. Furthermore, through the study of emotional labor-related courses, teachers can gain knowledge and practical skills, integrating theory with practice to improve work performance.

### 6.6 Reasonably arrange activities to create a positive work atmosphere

A conducive teaching environment and atmosphere play a crucial role in facilitating teachers’ emotional expression. Therefore, it is essential to create comfortable workplaces and environments, establish good work and teaching order, and foster harmonious internal relationships. This includes fostering harmonious relationships between management and teachers, teachers and students, and teachers and society. It is important to empower teachers to take the lead in their work, establish a relaxed work atmosphere, and maintain good professional ethics. Clear work tasks and objectives should be outlined, and excessive pressure on teachers should be avoided. Regularly organizing entertainment or sports activities, encouraging teachers to pursue hobbies and interests, and fostering a relaxed and comfortable atmosphere can help release emotions, thereby continuously improving work performance.

## 7 Research contributions and limitations

### 7.1 Research contributions

#### 7.1.1 Theoretical contribution

The main contribution and innovation of this study lies in revealing the relationship between emotional labor and relational performance of university teachers, further expanding the extension of emotional event theory, and providing a theoretical basis for university managers to improve teachers’ emotional labor and job performance. The theoretical contributions are specifically manifested in the following three aspects: ① enriching the research on the impact mechanism of teachers’ emotional labor on work adaptation in the theory of emotional events and supplementing the lack of research on teachers’ emotional labor and work adaptation under this theory; ② beneficial for expanding the academic community’s understanding of the horizontal and vertical relationship between emotional event theory and teacher job performance; ③ further demonstrated the applicability of the relationship between emotional event theory and teacher job performance.

#### 7.1.2 Practical contribution

The cross-sectional and longitudinal study on the relationship between teacher emotional labor and job performance has the following practical significance: ① previous research has mostly explored the negative outcomes of teacher emotional labor, with less attention paid to its positive functions. Exploring the positive functions of teachers’ emotional labor is beneficial for teachers to recognize the significance of emotional labor, thus better implementing emotional labor and promoting their educational and teaching work; ② understanding the development trend and influencing factors of job performance can provide new methods and paths for scientific prevention and effective intervention of emotional labor affecting job performance.

### 7.2 Limitations of the study

There are still shortcomings in the research object and content of this study, and further research is needed to improve them.

Firstly, the sample size and scope of this study are not comprehensive enough, which to some extent affects the external validity of the research. Subsequent research can investigate a wider range of regions to further test the conclusions of this study.

Secondly, there are issues with the small sample size and concentrated sample sources in this study. Future research needs to further expand the sampling scope or focus on remote areas, teaching experience, school nature, and other aspects to understand their current situation and differences.

Finally, based on existing research and theories, this study investigates the impact of teacher emotional labor on job performance, but the mechanism of teacher emotional labor explored is still relatively simple. Further research will consider other variables to comprehensively explore the impact mechanism of teachers’ emotional labor on job performance.

## 8 Epilog

In summary, based on the empirical investigation of emotional labor and work performance, this study identifies specific mechanisms through which emotional labor affects university teachers’ work performance. Therefore, it is proposed that universities should focus on teachers’ emotional labor characteristics, constructing an environment and management mechanism that enhances university teachers’ emotions, ultimately leading to improvements in their work performance.

## Data Availability

The original contributions presented in the study are included in the article/supplementary material, further inquiries can be directed to the corresponding author.
